# Association of Incident Delirium With Short-term Mortality in Adults With Critical Illness Receiving Mechanical Ventilation

**DOI:** 10.1001/jamanetworkopen.2022.35339

**Published:** 2022-10-07

**Authors:** Hsiu-Ching Li, Tony Yu-Chang Yeh, Yu-Chung Wei, Shih-Chi Ku, Yu-Juan Xu, Cheryl Chia-Hui Chen, Sharon Inouye, Leanne M. Boehm

**Affiliations:** 1 Department of Nursing, Chang Gung University of Science and Technology, Taoyuan, Taiwan; 2 Department of Anaesthesiology, National Taiwan University College of Medicine and National Taiwan University Hospital, Taipei, Taiwan; 3 Graduate Institute of Statistics and Information Science, National Changhua University of Education, Changhua, Taiwan; 4 Department of Internal Medicine, National Taiwan University College of Medicine and National Taiwan University Hospital, Taipei, Taiwan; 5 Department of Nursing, National Taiwan University College of Medicine and National Taiwan University Hospital, Taipei, Taiwan; 6 Department of Medicine, Beth Israel Deaconess Medical Center, Harvard Medical School, Boston, Massachusetts; 7 Marcus Institute for Aging Research, Hebrew Senior Life, Boston, Massachusetts; 8 Associate Editor, *JAMA Network Open*; 9 Vanderbilt University School of Nursing, Nashville, Tennessee; 10 Critical Illness, Brain dysfunction, and Survivorship Center, Vanderbilt University Medical Center, Nashville, Tennessee

## Abstract

**Importance:**

Intensive care unit (ICU)–acquired delirium and/or coma have consequences for patient outcomes. However, contradictory findings exist, especially when considering short-term (ie, in-hospital) mortality and length of stay (LOS).

**Objective:**

To assess whether incident delirium, days of delirium, days of coma, and delirium- and coma-free days (DCFDs) are associated with 14-day mortality, in-hospital mortality, and hospital LOS among patients with critical illness receiving mechanical ventilation.

**Design, Setting, and Participants:**

This single-center prospective cohort study was conducted in 6 ICUs of a university-affiliated tertiary hospital in Taiwan. A total of 267 delirium-free patients (aged ≥20 years) with critical illness receiving mechanical ventilation were consecutively enrolled from August 14, 2018, to October 1, 2020.

**Exposures:**

Participants were assessed daily for the development of delirium and coma status over 14 days (or until death or ICU discharge) using the Confusion Assessment Method for the Intensive Care Unit and the Richmond Agitation-Sedation Scale, respectively.

**Main Outcomes and Measures:**

Mortality rates (14-day and in-hospital) and hospital LOS using electronic health records.

**Results:**

Of 267 participants (median [IQR] age, 65.9 [57.4-75.1] years; 171 men [64.0%]; all of Taiwanese ethnicity), 149 patients (55.8%) developed delirium for a median (IQR) of 3.0 (1.0-5.0) days at some point during their first 14 days of ICU stay, and 105 patients (39.3%) had coma episodes also lasting for a median (IQR) of 3.0 (1.0-5.0) days. The 14-day and in-hospital mortality rates were 18.0% (48 patients) and 42.1% (112 of 266 patients [1 patient withdrew from the study]), respectively. The incidence and days of delirium were not associated with either 14-day mortality (incident delirium: adjusted hazard ratio [aHR], 1.37; 95% CI, 0.69-2.72; delirium by day: aHR, 1.00; 95% CI, 0.91-1.10) or in-hospital mortality (incident delirium: aHR, 1.00; 95% CI, 0.64-1.55; delirium by day: aHR, 1.02; 95% CI, 0.97-1.07), whereas days spent in coma were associated with an increased hazard of dying during a given 14-day period (aHR, 1.16; 95% CI, 1.10-1.22) and during hospitalization (aHR, 1.10; 95% CI, 1.06-1.14). The number of DCFDs was a protective factor; for each additional DCFD, the risk of dying during the 14-day period was reduced by 11% (aHR, 0.89; 95% CI, 0.84-0.94), and the risk of dying during hospitalization was reduced by 7% (aHR, 0.93; 95% CI, 0.90-0.97). Incident delirium was associated with longer hospital stays (adjusted β = 10.80; 95% CI, 0.53-21.08) when compared with no incident delirium.

**Conclusions and Relevance:**

In this study, despite prolonged LOS, ICU delirium was not associated with short-term mortality. However, DCFDs were associated with a lower risk of dying, suggesting that future research and intervention implementation should refocus on maximizing DCFDs to potentially improve the survival of patients receiving mechanical ventilation.

## Key Points

**Question:**

Are incident delirium, days of delirium, days of coma, and delirium- and coma-free days (DCFDs) associated with short-term mortality and hospital length of stay?

**Findings:**

In this prospective cohort study of 267 patients with critical illness receiving mechanical ventilation, delirium was not associated with short-term mortality, despite being associated with longer hospital stays. Days spent in coma were associated with an increased hazard of dying within a given 14-day period and during hospitalization, whereas the number of DCFDs was a protective factor.

**Meaning:**

The findings of this study support adoption of interventions (eg, the Hospital Elder Life Program and the ABCDEF bundle) to maximize DCFDs, thereby potentially increasing in-hospital survival of patients receiving mechanical ventilation.

## Teaser

This cohort study assesses the association of incident delirium, days of delirium, days of coma, and delirium- and coma-free days with short-term mortality and hospital length of stay among adult patients with critical illness receiving mechanical ventilation.

## Introduction

Delirium, a form of acute brain dysfunction, occurs in up to 80% of patients receiving mechanical ventilation in the intensive care unit (ICU).^1^ Delirium occurring in the ICU is associated with increased in-hospital mortality, longer duration of mechanical ventilation, and long-term cognitive impairment.^2,3,4,5,6,7^ There are conflicting findings on whether ICU delirium (both incident delirium and days of delirium) is associated with 28- or 90-day short-term mortality,^8^ especially after adjusting for all substantial competing risks, including age, severity of illness, presence of sepsis, mechanical ventilation use, and haloperidol use. Delirium is also associated with longer hospital length of stay (LOS),^2,9^ but there are inconsistent findings for the association between ICU delirium and LOS.^3,10,11^

As new guidelines providing recommendations for the prevention and management of agitation, sedation, and delirium^12^ are adopted into critical care practice, the association of ICU delirium with short-term mortality and LOS may change. In addition, coma (ie, a Richmond Agitation-Sedation Scale [RASS]^13^ score of −4 or –5) has been suggested to be a greater risk factor than ICU delirium for short-term mortality.^8^ In general, the delirium-mortality association (up to 18 months) has been confined to patients with coma.^14^ As a negative surrogate, delirium- and coma-free days (DCFDs) provide an estimate of the duration of normal brain function and have been used as an outcome in previous high-impact studies.^15,16,17^

In hospitals in Taiwan, acute brain dysfunction is presenting as a hidden and increasing epidemic, especially in the ICU setting. In a national effort, Taiwan released guidelines to facilitate the adoption of protocolized delirium monitoring and other evidence-based delirium interventions.^18^ Thus, we conducted a prospective cohort study to evaluate the association of incident delirium, days of delirium, days of coma, and DCFDs with short-term (14-day and in-hospital) mortality and hospital LOS to verify the relevant outcomes for delirium prevention and management.

## Methods

This prospective cohort study of patients receiving mechanical ventilation was approved by the National Taiwan University Hospital Research Ethics Committee. Written informed consent was obtained from all participants or their surrogates. The study followed the Strengthening the Reporting of Observational Studies in Epidemiology (STROBE) reporting guideline for cohort studies.

### Setting and Sample

From August 14, 2018, to October 1, 2020, we enrolled consecutive adult patients (aged ≥20 years) admitted to 6 medical ICUs (chest, cardiac, gastrointestinal, or mixed medical) of a university-affiliated tertiary hospital in Taiwan. Qualified patients for the study included those who were delirium or coma free and received mechanical ventilation with an expected ICU stay exceeding 24 hours. Patients were excluded if they (1) could not respond to the questions or evaluation protocol (eg, moderate dementia defined as a Clinical Dementia Rating [CDR] scale^19^ score of ≥2], psychotic disease, or severe hearing impairment), (2) were confined to bed for a prolonged period before index hospitalization (based on our study aim of monitoring patient recovery through activities of daily living [ADL]), or (3) were placed on contact and droplet precautions (eg, for open tuberculosis or COVID-19). Exclusion criteria, defined a priori, are shown in the patient flow diagram (Figure).

**Figure.**
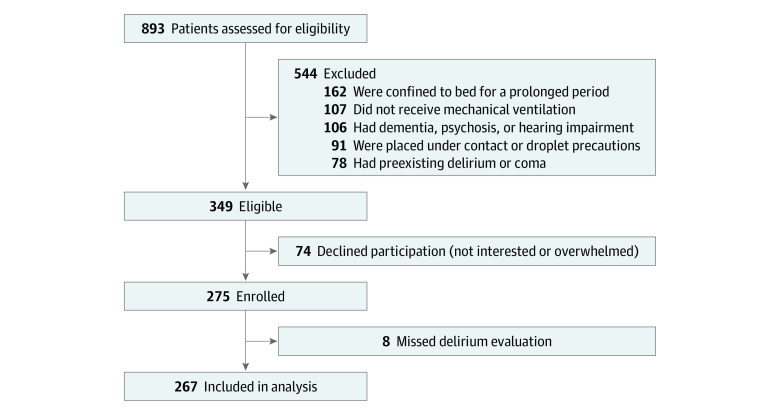
Study Flow Diagram

### Study Procedures

Two trained research nurses (H.C.L. and Y.J.X.) evaluated participants by collecting cohort demographic (age in years and sex) and clinical characteristics. The Acute Physiology and Chronic Health Evaluation II^20^ (APACHE II; score range, 0-71, with higher scores indicating greater illness severity) was applied using the most abnormal values obtained during the first 24 hours of ICU stay. The Charlson Comorbidity Index (CCI)^21^ (score range, 0-31, with higher scores indicating more comorbid burden) was calculated to represent the sum of weighted preexisting comorbid conditions at ICU admission. Sepsis (yes or no) was defined as a Sequential Organ Failure Assessment (SOFA)^22^ score of 2 or higher at ICU admission. The SOFA evaluates 6 organ systems, with a total score ranging from 0 (best) to 24 (worst).^22^

The research nurses also rated the presence and extent of dementia using the CDR scale^19^ (a 5-point scale measuring 6 domains, with level 0 indicating no dementia and levels 0.5, 1.0, or 1.5 indicating mild dementia). Participant ADL function before the index hospitalization was assessed using the Barthel Index.^23,24^ The Barthel Index measures independence in feeding, bathing, grooming, dressing, bowel or bladder function, toilet use, transfers, mobility, and climbing stairs; scores were classified into 3 functional categories: severely to fully dependent (0-60 points), slightly to moderately dependent (61-99 points), and fully independent (100 points)).^23,24^

The ICU admission diagnoses (eg, respiratory failure, cardiac emergency, noncardiogenic shock, and others) were abstracted from medical records. Delirium and coma were assessed once daily by trained research nurses until ICU discharge or for a maximum of 14 days, whichever occurred first. All research nurses had at least 1 year of ICU experience and received 1:1 training on delirium evaluation from 2 intensivists (T.Y.C.Y. and S.C.K.), an experienced nurse practitioner (C.C.H.C.), and a psychiatrist. Nurse ICU delirium screening was calibrated with an experienced psychiatrist during the study.

### Variables and Measures

#### Exposures

Delirium (yes or no) was measured using the Confusion Assessment Method for the Intensive Care Unit (CAM-ICU)^25^ partnered with the RASS to measure the level of consciousness. Coma was defined as a RASS score of −4 or −5, making those participants ineligible for CAM-ICU screening.^13^ We considered the fact that brain function fluctuates during an ICU stay, contributing to different outcomes; thus, the data were codified into delirium days, coma days, and DCFDs. We did not exclude individuals with persistent coma from our analyses (ie, RASS score of −4 or −5 at every evaluation until death or 14 days in the ICU). Therefore, the measure of coma days reflected the cumulative consequences of the comatose state. Delirium- and coma-free days (ie, the number of days during the study period [maximum of 14] during which the patient was alive without delirium or coma associated with any cause) provided an estimate of the duration of normal brain function. Any remaining study days were recorded as 0 for patients who died or were discharged from the ICU before day 14 (eg, DCFDs were recorded as 1 if the participant was free of delirium and coma for 1 day, had delirium for 3 days, and died the next day). Rather than recording 0 DCFDs when patients died within the study period,^17^ we counted the DCFDs the same way regardless of whether participants died within the study period.

#### Outcomes

Mortality (ie, date of death) was evaluated at 14 days and at the time of hospital discharge. Hospital and ICU LOS days were abstracted from the electronic health records. Both mortality and LOS data were collected by an independent outcome assessor blinded to the study hypothesis and delirium assessment results.

### Statistical Analysis

All data were entered into a Research Electronic Data Capture (REDCap) database, and analyses were performed using statistical packages of IBM SPSS Statistics software, version 22 (IBM Corporation), with all tests being 2-tailed and *P* < .05 considered significant. Sample characteristics were reported as numbers with percentages or medians with IQRs. Survival time associations of incident delirium, days of delirium, days of coma, and DCFDs with 14-day and in-hospital mortality were estimated using Cox proportional hazards regression analysis. Because the proportionality of the hazards is an important assumption of Cox proportional hazards analysis, we evaluated the assumption using scaled Schoenfeld residuals.^26^ Once this assumption was met, we reported our results as adjusted hazard ratios (aHRs) with 95% CIs. Five confounders (age, baseline APACHE II score, CCI score, presence of sepsis [SOFA score ≥2], and mechanical ventilation days) known to be associated with mortality in adults with critical illness were identified a priori and subsequently forced into each model. Multivariable linear regression analysis was used to assess hospital LOS.

## Results

Among 267 participants, the median (IQR) age was 65.9 (57.4-75.1) years; 140 participants (52.4%) were 65 years and older, 171 (64.0%) were male, and 96 (36.0%) were female (Table 1). All participants were of Taiwanese ethnicity. Most participants (260 [97.4%]) had scores of 0 on the CDR, suggesting no evidence of dementia. At ICU admission, the median (IQR) CCI score was 3 (2-6), with a median (IQR) APACHE II score of 23 (17-29), indicating moderate comorbidity and relatively high severity of illness. Overall, 257 participants (96.3%) presented with sepsis, with a median (IQR) SOFA score of 8 (6-12). Participant baseline functional status before the index hospitalization was generally independent; 174 participants (65.2%) had scores of 100, indicating they were fully independent with ADL, whereas 60 participants (22.5%) had slight to moderate dependence, and the remaining 33 (12.4%) had severe to full dependence. Indications for ICU admission varied. Acute respiratory failure (174 participants [65.2%]) and noncardiogenic shock (66 participants [24.7%]) were the most common admission diagnoses. All participants received mechanical ventilation for a median (IQR) duration of 5.9 (3.2-10.3) days.

**Table 1.** Characteristics of Cohort

Characteristic	Participants, No./total No. (%) (N = 267)
Age, median (IQR), y	65.9 (57.4-75.1)
Sex	
Male	171/267 (64.0)
Female	96/267 (36.0)
ADL functional status before index hospitalization^a^	
Severely to fully dependent	33/267 (12.4)
Slightly to moderately dependent	60/267 (22.5)
Fully independent	174/267 (65.2)
Diagnosis at ICU admission	
Acute respiratory failure	174/267 (65.2)
Noncardiogenic shock	66/267 (24.7)
Cardiac emergency	24/267 (9.0)
Other^b^	3/267 (1.1)
CDR score of 0	260/267 (97.4)
CCI score, median (IQR)	3 (2-6)
24-h APACHE II score, median (IQR)	23 (17-29)
Sepsis^c^	257/267 (96.3)
SOFA score at admission, median (IQR)	8 (6-12)
Duration of mechanical ventilation, median (IQR), d	5.9 (3.2-10.3)
Ever had delirium	149/267 (55.8)
Delirium days, median (IQR)^d^	3.0 (1.0-5.0)
Ever in coma	105/267 (39.3)
Coma days, median (IQR)^d^	3.0 (1.0-5.0)
Delirium-free and coma-free days, median (IQR)^d^	3.0 (1.0-6.0)
ICU LOS, median (IQR), d	9.0 (5.0-15.0)
14-d Mortality	48/267 (18.0)
In-hospital mortality^e^	112/266 (42.1)
Delirium group	67/148 (45.3)
Nondelirium group	30/98 (30.6)
Persistent coma group	15/20 (75.0)
Hospital LOS, median (IQR), d^b^	25.0 (14.0-49.3)

Abbreviations: ADL, activities of daily living; APACHE II, Acute Physiology and Chronic Health Evaluation II; CCI, Charlson Comorbidity Index; CDR, Clinical Dementia Rating; LOS, length of stay; SOFA, Sequential Organ Failure Assessment.

^a^ Based on Barthel Index score, with 0-60 indicating severely to fully dependent, 61-99 indicating slightly to moderately dependent, and 100 indicating fully independent.

^b^ Includes drug overdoses, epilepsy, and diabetic ketoacidosis.

^c^ SOFA score of 2 or higher.

^d^ Maximum of 14 days.

^e^ One participant withdrew from the study.

During the first 14 days in the ICU, 149 participants (55.8%) developed delirium, and 105 participants (39.3%) spent 1 or more days with coma (Table 1). The median (IQR) duration for both delirium and coma was 3.0 (1.0-5.0) days. For the entirety of the sample, the median (IQR) number of DCFDs was 3.0 (1.0-6.0). Overall mortality was substantial; 48 participants (18.0%) died within 14 days of ICU admission, and 112 of 266 participants (42.1%; 1 participant withdrew from the study) died during hospitalization (77 died in the ICU, and 35 died in the hospital ward). The median (IQR) ICU LOS was 9.0 (5.0-15.0) days, and the median (IQR) hospital LOS was 25.0 (14.0-49.3) days. Notably, although 12 participants opted out of the study during ICU or hospital discharge, 11 agreed to release their deidentified delirium evaluations and hospital outcome data for research analyses. The main reason for study withdrawal was being overwhelmed and unable to commit to a 1-year follow-up.

### Association of ICU Delirium With Short-term Mortality

The Cox proportional hazards regression analysis reavealed that neither incident delirium nor days of delirium were associated with 14-day mortality (incident delirium: aHR, 1.37; 95% CI, 0.69-2.72; delirium by day: aHR, 1.00; 95% CI, 0.91-1.10) or in-hospital mortality (incident delirium: aHR, 1.00; 95% CI, 0.64-1.55; delirium by day: aHR, 1.02; 95% CI, 0.97-1.07) (Table 2). Days of coma were associated with a significantly higher adjusted hazard ratio for 14-day mortality (aHR, 1.16; 95% CI, 1.10-1.22) and in-hospital mortality (aHR, 1.10; 95% CI, 1.06-1.14) after adjusting for participant age, APACHE II scores, CCI scores, presence of sepsis, and mechanical ventilation days. In contrast, DCFDs were independently associated with a lower risk of dying at both 14 days and during hospitalization; with each additional DCFD, the risk of dying was reduced by 11% at 14 days (aHR, 0.89; 95% CI, 0.84-0.94) and 7% during hospitalization (aHR, 0.93; 95% CI, 0.90-0.97), independent of age, disease severity, comorbidity, sepsis, and mechanical ventilation days.

**Table 2.** Association of Delirium, Coma, and DCFDs With Short-term Mortality

Variable	HR (95% CI)			
	14-d Mortality (N = 267)		In-hospital mortality (n = 266)^a^	
	Unadjusted	Adjusted^b^	Unadjusted	Adjusted^b^
Incident delirium	1.03 (0.53-1.99)	1.37 (0.69-2.72)	1.08 (0.70-1.66)	1.00 (0.64-1.55)
Delirium by day	0.82 (0.70-0.96)	1.00 (0.91-1.10)	1.02 (0.97-1.07)	1.02 (0.97-1.07)
Coma by day	1.10 (1.02-1.18)	1.16 (1.10-1.22)	1.09 (1.05-1.13)	1.10 (1.06-1.14)
DCFDs	0.90 (0.86-1.95)	0.89 (0.84-0.94)	0.93 (0.90-0.97)	0.93 (0.90-0.97)

Abbreviations: DCFDs, delirium- and coma-free days; HR, hazard ratio.

^a^ One participant withdrew from the study.

^b^ All models were adjusted for age, Acute Physiology and Chronic Health Evaluation II score, Charlson Comorbidity Index score, presence of sepsis (Sequential Organ Failure Assessment score of ≥2), and mechanical ventilation days.

To examine whether including participants with persistent coma in the analyses altered the findings, we reanalyzed the data by excluding participants with persistent coma, as performed in most previous studies^4,9^ (eTable 1 in the Supplement). We found the associations between days of coma and mortality were similar; aHRs slightly increased to 1.25 (95% CI, 1.16-1.35) for 14-day mortality and 1.11 (95% CI, 1.05-1.18) for in-hospital mortality. Delirium- and coma-free days were also associated with a lower risk of dying at both 14 days (aHR, 0.89; 95% CI, 0.83-0.95) and during hospitalization (aHR, 0.94; 95% CI, 0.91-0.98).

### Association of ICU Delirium With Hospital LOS

Neither days of delirium (adjusted β = 0.37; 95% CI, −0.95 to 1.69), days of coma (adjusted β = −0.81; 95% CI, −1.94 to 0.31), nor DCFDs (adjusted β = 0.25; 95% CI, −0.66 to 1.15) were associated with hospital LOS (Table 3). Only incident delirium was independently associated with increases in hospital LOS (adjusted β = 10.80; 95% CI, 0.53-21.08).

**Table 3.** Association of Delirium, Coma, and DCFDs With Hospital Length of Stay

Length of hospital stay	β (95% CI)^a^	
	Unadjusted	Adjusted^b^
Incident delirium	12.74 (2.49 to 22.99)	10.80 (0.53 to 21.08)
Delirium by day	0.48 (–0.80 to 1.77)	0.37 (–0.95 to 1.69)
Coma by day	–0.31 (–1.40 to 0.78)	–0.81 (–1.94 to 0.31)
DCFDs	–0.10 (–0.98 to 0.77)	0.25 (–0.66 to 1.15)

Abbreviation: DCFDs, delirium- and coma-free days.

^a^ Among 266 participants (1 participant withdrew from the study).

^b^ All models were adjusted for age, Acute Physiology and Chronic Health Evaluation II score, Charlson Comorbidity Index score, presence of sepsis (Sequential Organ Failure Assessment score of ≥2), and mechanical ventilation days.

Because we suspected that increased mortality among participants with coma (or persistent coma) masked the association with LOS, we reanalyzed the data. First, we excluded participants with persistent coma (n = 246) and found that results remained similar; only incident delirium was significantly associated with longer LOS (adjusted β = 10.80; 95% CI, 0.53-21.08) (eTable 2 in the Supplement). Second, we restricted the analysis to survivors of the hospital stay (n = 154) and found a similar result; only incident delirium was associated with prolonged LOS (adjusted β = 18.57; 95% CI, 3.97-33.16), whereas all other factors were not significantly associated with LOS (eTable 3 in the Supplement). However, the confidence intervals CIs for the LOS β estimates were wide, suggesting that these results should be interpreted with caution.

## Discussion

In this prospective cohort study of 267 patients receiving mechanical ventilation, we found delirium and coma were common during the first 14 ICU days. Neither incident delirium nor days of delirium were associated with risk of 14-day or in-hospital mortality, but the overall 14-day mortality hazard increased with each coma day added. Notably, the risks of dying within 14 days or during hospitalization were incrementally lower with each additional DCFD. Despite protocolized delirium monitoring and care in the participating ICUs during the study period, there was room to improve processes to maximize DCFDs and thus short-term mortality in the ICUs under study.

In our study, delirium alone was not associated with short-term mortality, and the number of days spent in coma was significantly associated with the risk of 14-day and in-hospital mortality. These results build on those of a previous Dutch study,^8^ which reported that neither incident delirium nor days spent with delirium were associated with short-term (28- and 90-day) mortality, but days spent in coma increased the odds of 28- and 90-day mortality. There are 3 possible explanations for the contradictory findings of delirium-associated mortality in the literature.^2,3,4,5,27^ First, patients who presented with delirium before ICU admission were excluded from our study and the Dutch study (ie, only patients with incident delirium were included). Second, improvements in ICU care may have resulted in more recent cohort studies of patients with critical illness reporting a lower delirium incidence, with an increasing pattern of negative findings for short-term mortality after ICU delirium compared with previous investigations.^28,29^ Third, the current practice of excluding individuals with coma (RASS score of −4 or −5) from delirium screening may result in potential underestimation of the rate of delirium and overestimation of the role of coma within the sample. We acknowledge that delirium and coma exist on a spectrum of acute brain dysfunction. Thus, the traditional approach of separating the 2 variables when evaluating their individual associations with mortality might not be clinically warranted. It may also inadequately account for the fact that they are on a continuum.

We found that DCFDs were associated with lower odds of 14-day and in-hospital mortality. Because delirium and coma are closely related and both are associated with adverse outcomes, targeting improvement in DCFDs as the basis of outcomes and quality measures in ICUs to improve short-term survival is clinically warranted. As a means to facilitate the implementation of recent critical care guidelines to improve outcomes, bundled nonpharmacological interventions have been increasingly studied.^30^ The Hospital Elder Life Program (HELP) has been reported to be effective in preventing delirium in hospitalized older adults.^31^ The HELP provides a multiple component protocol targeting risk factors for delirium. The ABCDEF (assess, prevent and manage pain; both spontaneous awakening and breathing trials; choice of analgesia or sedation; delirium: assess, prevent, and manage; early mobility; and family engagement and empowerment) bundle is a multicomponent guideline-recommended model that coordinates the people, processes, and technologies in the ICU to result in patients who are awake, cognitively engaged, and physically active.^12,32,33^ Despite its efficacy in reducing next-day delirium and coma, the bundle is still underused in ICUs worldwide.^34^ The findings of this study support bundle adoption to maximize DCFDs, thus potentially increasing in-hospital survival of patients receiving mechanical ventilation.

### Limitations

This study has several limitations. First, as in any observational study, this study is not designed to assess causality. Moreover, we acknowledge the limitation of using DCFDs to estimate in-hospital mortality. Similar to ventilator-free days, such failure-free day concepts, which combine survival and ventilation duration or delirium and coma (in the case of this study), penalize nonsurvivors.^35^ Thus, the finding of protective benefit should be interpreted with caution and replicated by more independent assessments.

Second, we did not measure the timing of delirium evaluation and daily sedation cessation. Not measuring those factors could have overestimated delirium incidence while participants were receiving sedative medications (ie, rapidly reversible sedative-associated delirium might have been included). In addition, delirium and coma incidence and duration may be underestimated because we performed once-daily delirium assessment for 14 days (or until death or ICU discharge), potentially missing rapid fluctuations in mental status. We specifically chose a 14-day ICU delirium evaluation period because it represents the best balance for covering the dynamic ICU course to gain important data while maximizing resource use, given the median (IQR) LOS in the participating ICUs was 9.0 (5.0-15.0) days. Findings from a previous study^36^ supported a combination of patient assessment, medical record review, and medication administration record review to improve the sensitivity of delirium identification for research purposes. Researchers in the Netherlands have also identified a 5-step algorithm to classify mental status based on the CAM-ICU.^37^ Future studies might incorporate those tools to enhance the sensitivity of delirium identification.

Third, no data on sedative or antipsychotic medication use were collected, and we generally included confounding factors at baseline. Because many factors can change over time (eg, APACHE II score or sepsis may worsen or improve later in the ICU course), residual confounding in the associations of delirium and coma with mortality cannot be excluded. Lack of use of time-varying cofounders is also acknowledged.

Fourth, this study was conducted in a single center, and the exclusion criteria of a CDR score of 2 or higher and preexisting delirium at ICU admission likely resulted in a cohort with better cognitive reserve (97.4% of participants had scores of 0 on the CDR); thus, the results are restricted to those without preexisting acute or chronic cognitive impairment and may not generalize to all ICU populations. Moreover, although we did not enroll individuals with COVID-19, our study spans the COVID-19 period from March to October 2020, which may have impacted the study results and mortality rates.

Fifth, we did not collect data on delirium subtypes and are thus unable to verify previous findings that only mixed delirium (not rapidly reversible, hyperactive, or hypoactive delirium) is associated with mortality.^38^ Future studies with larger samples from representative sites and with careful design in delirium evaluation are warranted to detangle the complex interplay between delirium (or motoric subtypes) and coma and, most important, to examine whether maximizing ICU DCFDs would improve hospital survival.

## Conclusions

This cohort study found that delirium in patients with critical illness receiving mechanical ventilation was not consistently associated with short-term (in-hospital or 14 days after ICU admission) mortality. Nonetheless, ICU DCFDs may be a protective factor. With each additional DCFD, the risk of dying within 14 days or during hospitalization was independently reduced. Future research and intervention implementation might refocus on maximizing DCFDs, thus potentially improving the survival of patients receiving mechanical ventilation.

## Notes

Supplement. **eTable 1.** Analysis Excluding Patients With Persistent Coma: Association of Delirium, Coma, and Delirium- and Coma-Free Days With Mortality **eTable 2.** Analysis Excluding Patients With Persistent Coma: Association of Delirium, Coma, and Delirium- and Coma-Free Days With Hospital Length of Stay **eTable 3.** Analysis Restricted to Survivors: Association of Delirium, Coma, and Delirium- and Coma-Free Days With Hospital Length of Stay
