# Supplementary material for: Association of Incident Delirium With Short-term Mortality in Adults With Critical Illness Receiving Mechanical Ventilation
Source: JAMA Netw Open. 2022 Oct 7;5(10):e2235339. doi: 10.1001/jamanetworkopen.2022.35339 (PMC9547314; doi:10.1001/jamanetworkopen.2022.35339)
Supplement: Supplement. — eTable 1. Analysis Excluding Patients With Persistent Coma: Association of Delirium, Coma, and Delirium- and Coma-Free Days With Mortality eTable 2. Analysis Excluding Patients With Persistent Coma: Association of Delirium, Coma, and Delirium- and Coma-Free Days With Hospital Length of Stay eTable 3. Analysis Restricted to Survivors: Association of Delirium, Coma, and Delirium- and Coma-Free Days With Hospital Length of Stay [file jamanetwopen-e2235339-s001.pdf]

# Supplementary Online Content

Li HC, Yeh TYC, Wei YC, et al. Association of incident delirium with short-term mortality in adults with critical illness receiving mechanical ventilation. *JAMA Netw Open*. 2022;5(10):e2235339. doi:10.1001/jamanetworkopen.2022.35339

**eTable 1.** Analysis Excluding Patients With Persistent Coma: Association of Delirium, Coma, and Delirium- and Coma-Free Days With Mortality

**eTable 2.** Analysis Excluding Patients With Persistent Coma: Association of Delirium, Coma, and Delirium- and Coma-Free Days With Hospital Length of Stay

**eTable 3.** Analysis Restricted to Survivors: Association of Delirium, Coma, and Delirium- and Coma-Free Days With Hospital Length of Stay

This supplementary material has been provided by the authors to give readers additional information about their work.

© 2022 Li HC et al. *JAMA Network Open*.

**eTable 1.** Analysis Excluding Patients With Persistent Coma: Association of Delirium, Coma, and Delirium- and Coma-Free Days With Mortality

| Mortality | 14-Day Mortality (n=247) | | In-Hospital Mortality (n=246)[a] | |
|---|---|---|---|---|
| | Unadjusted HR (95% CI) | Adjusted HR (95% CI) | Unadjusted HR (95% CI) | Adjusted HR (95% CI) |
| Incident delirium | 1.03 (0.53 to 1.99) | 1.37 (0.69 to 2.72) | 1.08 (0.70 to 1.07) | 1.00 (0.64 to 1.55) |
| Delirium (by day) | 0.82 (0.70 to 0.96) | 1.00 (0.91 to 1.10) | 1.02 (0.97 to 1.07) | 1.02 (0.97 to 1.07) |
| Coma (by day) | 1.15 (1.10 to 1.21) | 1.25 (1.16 to 1.35) | 1.11 (1.05 to 1.16) | 1.11 (1.05 to 1.18) |
| Delirium-/Coma-free days | 0.91 (0.86 to 0.96) | 0.89 (0.83 to 0.95) | 0.94 (0.90 to 0.97) | 0.94 (0.91 to 0.98) |

All models were adjusted for age, acute physiology and chronic health evaluation II scores, Charlson Comorbidity Index scores, sepsis (sequential organ failure assessment score ≥ 2), and mechanical ventilation days.

[a]1 dropout.

© 2022 Li HC et al. *JAMA Network Open*.

**eTable 2.** Analysis Excluding Patients With Persistent Coma: Association of Delirium, Coma, and Delirium- and Coma-Free Days With Hospital Length of Stay (n = 246)

| **Length of Hospital Stay** | **All Sample (n=246)[a]** | |
|---|---|---|
| | **Unadjusted β (95% CI)** | **Adjusted β (95% CI)** |
| Incident delirium | 12.74 (2.49 to 22.99) | 10.80 (0.53 to 21.08) |
| Delirium (by day) | 0.48 (-0.80 to 1.77) | 0.37 (-0.95 to 1.69) |
| Coma (by day) | 0.18 (-1.50 to 1.85) | -0.95 (-2.72 to 0.82) |
| Delirium-/Coma-free days | -0.34 (-1.33 to 0.65) | 0.10 (-0.92 to 1.11) |

All models were adjusted for age, acute physiology and chronic health evaluation II scores, Charlson Comorbidity Index scores, sepsis (sequential organ failure assessment score ≥ 2), and mechanical ventilation days.

[a]1 dropout.

© 2022 Li HC et al. *JAMA Network Open*.

**eTable 3.** Analysis Restricted to Survivors: Association of Delirium, Coma, and Delirium- and Coma-Free Days With Hospital Length of Stay (n = 154)

| Length of Hospital Stay | Restricted to Survivors (n=154) | |
|---|---|---|
| | Unadjusted β (95% CI) | Adjusted β (95% CI) |
| Incident delirium | 20.33 (5.80 to 34.85) | 18.57 (3.97 to 33.16) |
| Delirium (by day) | 1.03 (-0.86 to 2.91) | 0.92 (-0.98 to 2.82) |
| Coma (by day) | 0.67 (-1.68 to 3.01) | -0.47 (-2.78 to 1.83) |
| Delirium-/Coma-free days | -0.92 (-2.41 to 0.56) | -0.39 (-1.88 to 1.11) |

All models were adjusted for age, acute physiology and chronic health evaluation II scores, Charlson Comorbidity Index scores, sepsis (sequential organ failure assessment score ≥ 2), and mechanical ventilation days.

© 2022 Li HC et al. *JAMA Network Open*.
